# Electrostatic Deposition of Large-Surface Graphene

**DOI:** 10.3390/ma11010116

**Published:** 2018-01-12

**Authors:** Charles Trudeau, Laura-Isabelle Dion-Bertrand, Sankha Mukherjee, Richard Martel, Sylvain G. Cloutier

**Affiliations:** 1 Department of Electrical Engineering, École de Technologie Supérieure, 1100 Notre-Dame Ouest, Montréal, QC H3C 1K3, Canada; charlestrudeau4@gmail.com; 2 R&D Department, Phonon Etc., 5795 Avenue de Gaspé, Montréal QC H2S 2X3, Canada; lidion.bertrand@photonetc.com; 3 Department of Mechanical Engineering, McGill University, 845 Sherbrook Ouest, Montréal QC H3A 0G4, Canada; mukherjeeatom@gmail.com; 4 Department of Chemistry, Université de Montreal, 2900 Édouard-Montpetit, Montréal QC H3C 3J7, Canada; r.martel@umontreal.ca

**Keywords:** graphene, electrostatic deposition, Raman spectroscopy

## Abstract

This work describes a method for electrostatic deposition of graphene over a large area using controlled electrostatic exfoliation from a Highly Ordered Pyrolytic Graphite (HOPG) block. Deposition over 130 × 130 µm^2^ with 96% coverage is achieved, which contrasts with sporadic micro-scale depositions of graphene with little control from previous works on electrostatic deposition. The deposition results are studied by Raman micro-spectroscopy and hyperspectral analysis using large fields of view to allow for the characterization of the whole deposition area. Results confirm that laser pre-patterning of the HOPG block prior to cleaving generates anchor points favoring a more homogeneous and defect-free HOPG surface, yielding larger and more uniform graphene depositions. We also demonstrate that a second patterning of the HOPG block just before exfoliation can yield features with precisely controlled geometries.

## 1. Introduction

A decade ago, graphene exfoliation from bulk graphite by repeated surface cleaving using household Scotch tape was changing the world we live-in [1,2,3,4]. Since then, massive worldwide efforts explored the unique physical properties of single-and few-layer grapheme [5,6,7,8,9,10,11,12], including its optical [10,13,14,15,16,17] and electrical [5,6,10,13,15,16,18,19,20,21,22,23,24,25,26,27,28,29] properties. Novel deposition methods have also generated significant interest due to growing demands for large-scale device integration. Several deposition techniques are now available, each offering key advantages and significant drawbacks [3,4,9,11,12,19,30,31,32,33,34,35,36]. For instance, chemical vapor deposition (CVD) is currently the leading approach to produce large-scale graphene. This method exploits the chemical reaction between methane and hydrogen gases with a metallic surface to grow graphene directly on the surface [30,31,32,33,34]. Currently, CVD is the preferred graphene deposition method since it produces the best quality graphene and allows for precise control over the size and shape of the deposition [30,31,32,33,34]. However, this method is expensive and the transfer process can be difficult to scale up, which can be problematic for large-scale integration with conventional complementary metal-oxide-semiconductor (CMOS) microelectronic processes. In contrast, electrostatic deposition techniques exploit electrostatic forces to exfoliate the loosened graphene layers from bulk graphitic material and transfer directly onto a given substrate. However, cleaving of the bulk graphitic material prior to deposition is essential to loosen the graphene sheets. This cleaving process is generally performed with household tape and generates small samples (few μm^2^) of grapheme [35,36]. Electrostatic exfoliation would seem suitable for large area transfer, but it has not yet been capable of producing graphene flakes more than a few microns in size [35,36]. Nevertheless, electrostatic deposition remains one of the cheapest and fastest graphene deposition methods. In this report, we demonstrate how large-scale deposition (130 μm × 130 μm) of graphene is achieved using controlled electrostatic exfoliation from a pre-patterned HOPG block. Results show the pre-patterning of the HOPG block plays a crucial role in achieving larger and more uniform graphene depositions. In the future, we believe this significant improvement in terms of quality and size of the deposited graphene could dramatically increase the use of this simple deposition process and accelerate the integration of graphene within automated industrial processes for a wide range of applications.

## 2. Results and Discussion

**Figure 1:**
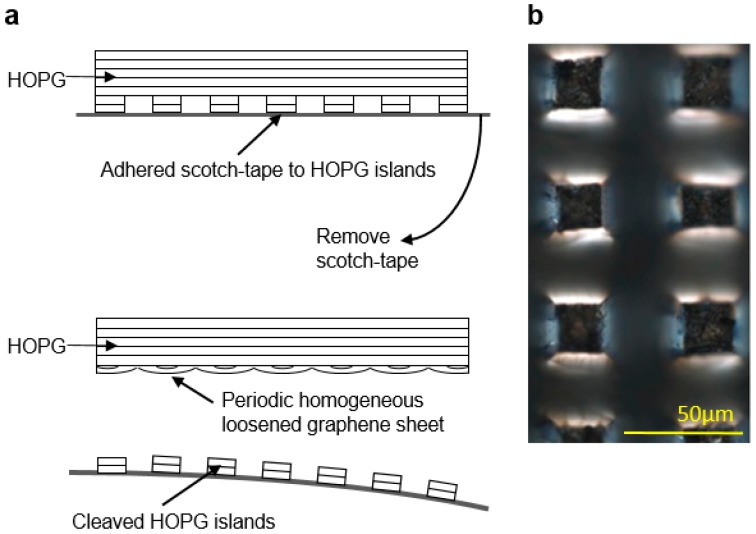
(**a**) Schematic representation of the proposed anchoring phenomenon resulting in large-scale deposition of graphene achieved in this work. (**b**) The pristine HOPG block is pre-patterned to create an array of 20 × 20 μm square islands using a UV laser marking system. The image was acquired using a LEXT OLS4000 laser microscope from Olympus.

Shown in Figure 1a is the schematic for the proposed improved cleaving process, the pristine HOPG block is pre-patterned to create an array of 20 × 20 μm^2^ square islands with 30 μm wide and 50 μm deep etched spacing between them using a UV laser marking system, the HOPG block with the etched features is shown in Figure 1b. After patterning, the HOPG block is cleaved using household tape to completely remove the etched features from the HOPG and provides a flat surface with less defects for deposition, as shown further in the results and discussion section.

**Figure 2:**
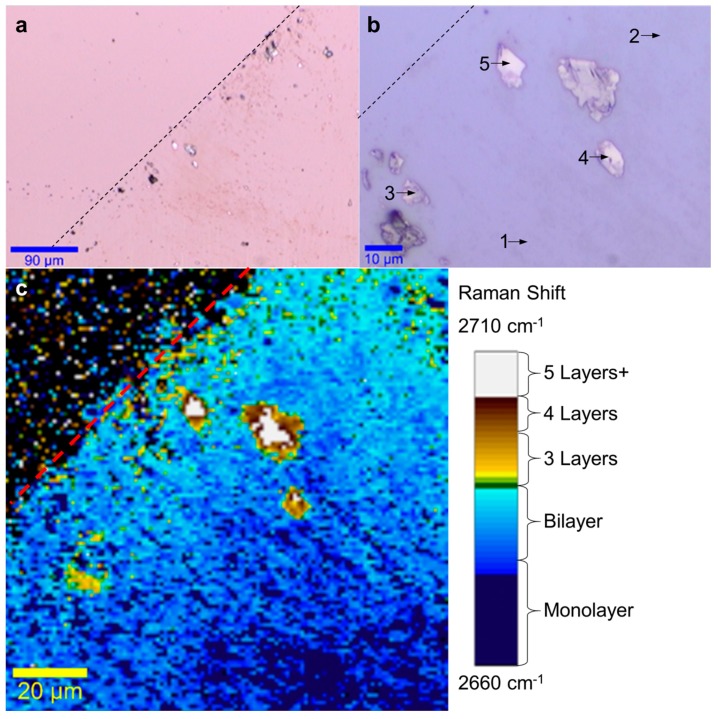
(**a**,**b**) Optical images at different magnifications, showing the edge of graphene deposition (dashed line), with arrows showing typical areas with specific numbers of graphene layers. (**c**) Raman image mapping the center of Gaussian curve fitted to the Raman 2D peak from 2660 cm^−1^ to 2710 cm^−1^.

**Figure 3:**
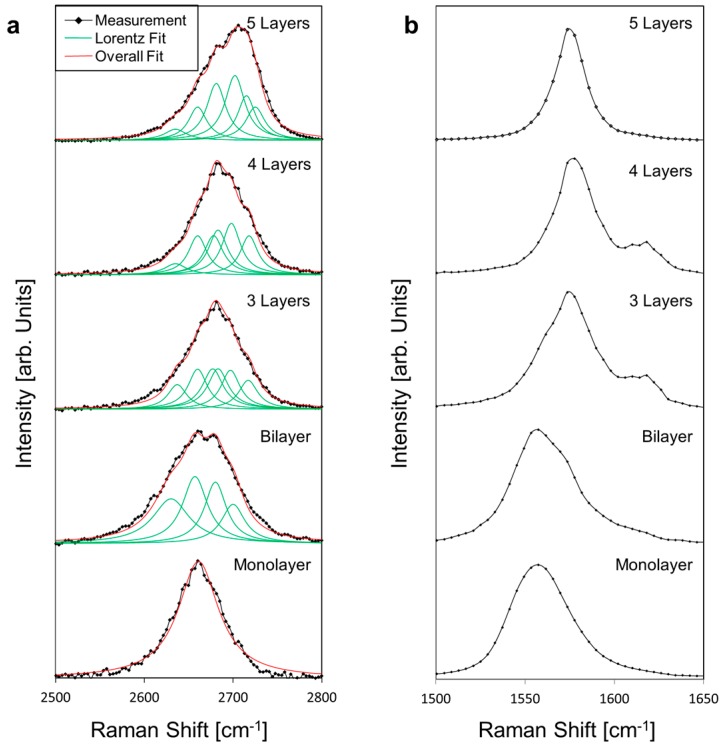
(**a**) Evolution of averaged and normalized Raman 2D-peak from monolayer to five-layer graphene from area specified in Figure 1b, with individual Lorentzian curves and the overall fit. (**b**) Averaged and normalized Raman G-peak evolution from monolayer to five-layer graphene from areas specified in Figure 1b.

Then, graphene is directly transferred onto a silicon wafer with a 1 µm-thick thermal oxide layer by using an exfoliation voltage of 3 kV. The complete details of the sample preparation and electrostatic deposition setup are provided in the methods section. The deposition results are shown in Figure 2. The optical images in Figure 2a,b respectively show the edge of the deposited graphene (dashed line) at 10× and 50×, while the Raman micro-spectroscopy scan in Figure 2c maps the 2D Raman peak position(between 2660 cm^−1^ and 2710 cm^−1^) for the same region. The 2D peak center is taken as the center of a single-fitted Gaussian obtained from the Raman data between 2550–2750 cm^−1^. In the optical images, single-layer graphene cannot be resolved due to its low light absorption [10,13], while some double-layered graphene features appear in Figure 2b and multi-layered graphene regions are easily differentiated from the oxidized-silicon substrate. In contrast, the 2D-band center Raman mapping shown in Figure 2c, clearly shows the full coverage of the deposited graphene and confirms the dominant monolayer and bilayer structure [37,38,39]. A color scale bar showing the 2D-band center with the estimated number of graphene layers has been added for layer counting. As shown further down, the Raman spectra acquired at the positions specified for each layer number (shown in Figure 3) are in agreement with theoretical and experimental evidence found in the literature [37,38,39]. From these images, it becomes clear that most of the deposited graphene is single-or double-layered with only a few multi-layered graphene flakes near the edge.

Meanwhile, Figure 3 shows the normalized Raman 2D-band peak evolution obtained experimentally from single-layer to five-layer graphene by averaging the specific areas identified in Figure 2b. These experimental results are consistent with theoretical predictions and experimental evidence [37,38,39]. Indeed, the splitting of the 2D peak as the number of graphene layer increases is shown by fitting the experimental curves with a set of Lorentzian curves. The full-width at half-maximum (FWHM) of the Lorentzian curves are measured between 24 cm^−1^ and 34 cm^−1^, with the exception of the single-layer Lorentzian curve measured at 55 cm^−1^ and the lowest Lorentzian used to fit the bi-layer curve at 50 cm^−1^. This deviation from the 24 cm^−1^ FWHM can be attributed to unintentional doping of charge carriers from the deposition method which is known to widen the 2D peak in single-layer graphene [40,41,42]. These values are summarized in Table 1. For comparison, Figure 3b shows the normalized Raman G-band for mono- to five-layer graphene obtained from the same areas. To obtain the values of the G peak center, a single Gaussian is used to fit the peak around 1500–1600 cm^−1^ to avoid any effect from the D’ shoulder (1620 cm^−1^). The variation of the G peak position is found to be fairly large, especially for mono- and two-layer graphene, this also suggests edge effects and excess charge carriers from the deposition method [41,42]. The large variation of G peak observed could also be explained by unintentional doping and/or stress effects which can be relevant in single layer graphene, but less so for multilayer graphene [43,44].

**Figure 4:**
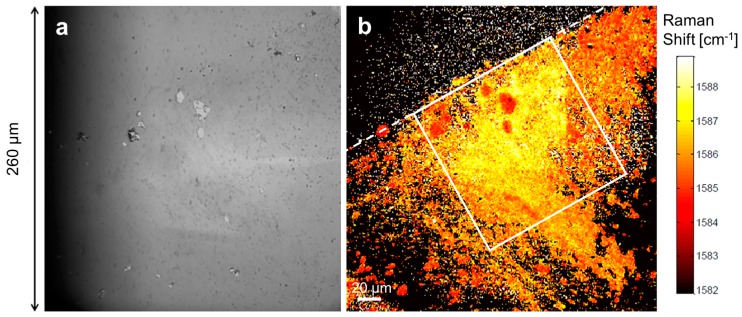
(**a**) White-light hyperspectral image with high field-of-view showing the edge of the deposition (dashed line). (**b**) Hyperspectral image of the full graphene deposition mapping the position of the highest intensity around the G peak (1500–1600 cm^−1^). The white box represents 130 μm × 130 μm. Acquired using RIMA^TM^ NANO (Photon Etc.).

Since the micro-Raman stage’s travel is restricting the scan size, hyperspectral analysis with a much larger field-of-view (FOV) can be used over the same deposition to fully appreciate the deposition scale. Figure 4a shows the wide-FOV white-light image of the deposition, much like the optical images shown in Figure 2a,b. While it clearly shows the multi-layered graphene regions, the single- and double-layered graphene are again much harder to observe directly. However, Figure 4b shows a hyperspectral mapping of the highest intensity wavenumber around the G peak [22,38]. Like in Figure 3b, a larger variation in the G-band peak position is seen for single- and double-layered graphene producing high color contrast. The same straight edge previously observed in the Raman images from Figure 2 is also clearly seen here. The white frame in Figure 4b delimits the 130 μm × 130 μm deposition area and includes a 92% ± 2% coverage with single-and double-layered graphene, 4% ± 1% with multi-layered (three-layer or more) graphene and only 4% ± 1% uncovered. SEM images of the same deposition area confirm the results obtained with the micro-Raman and hyperspectral analysis and are shown in the methods section.

**Figure 5:**
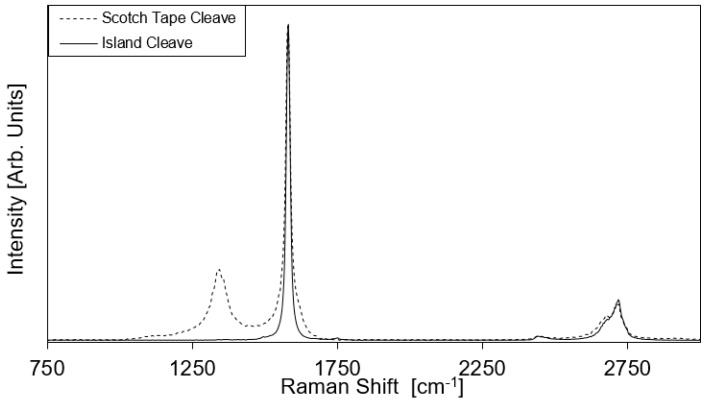
Average Raman spectrum of cleaved HOPG surface with and without pre-etching of graphene islands formed by laser pre-patterning. Graphs normalized for same G peak intensity.

Our study has clearly shown that the cleaving process is critical to achieving such large-scale depositions. Micro-Raman analysis of the cleaved HOPG surface with and without laser pre-patterning offers a direct assessment of the improved cleaving method and highlights the importance of the cleaving process. Indeed, the Raman spectra averaged over a 100 µm × 100 µm area of the HOPG block after regular scotch tape cleave with and without laser pre-patterning are compared in Figure 5. Results show no measurable D-band on the cleaved surface of the pre-patterned HOPG block after the complete removal of etched islands, indicating a surface with less defects [22,41,42] compared to the conventional scotch tape cleaving method with no pre-patterning. Since the large scale deposition observed here never occurs without the full removal of the etched graphene islands via scotch tape cleaving prior to electrostatic deposition, we can reasonably speculate that this success is the result of an anchoring effect of the patterned island on the bulk HOPG when they are removed. This anchoring effect appears to loosen the underlying graphene sheets periodically at each removed patterned island to create a very homogeneously loosened graphene sheet which can then be removed from the bulk HOPG with minimal tearing or damage, thus allowing for larger-scale graphene depositions. Figure 1a illustrate this proposed mechanism. All these results clearly show that the cleaving method used in the preparation of the block before the electrostatic exfoliation of graphene has a critical impact on the resulting depositions and that improvements in terms of homogeneity of the cleaving methods can significantly improve the overall deposition results. 

**Figure 6:**
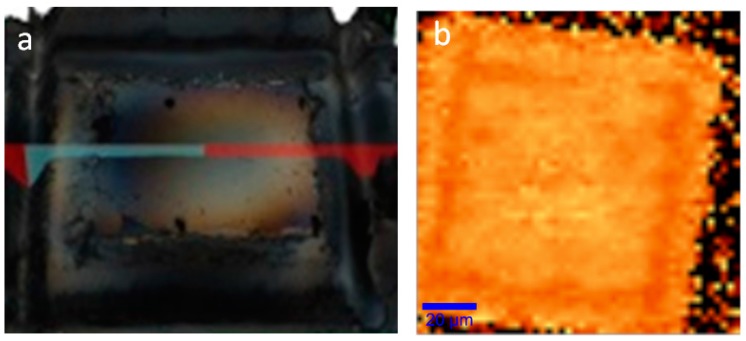
(**a**) To achieve more defined geometries, a second HOPG patterning step can be performed between the cleave and the electrostatic exfoliation using the UV laser marking system. Here, the HOPG was patterned with 90 μm × 90 μm square features and measured using a LEXT OLS4000 laser microscope from Olympus. (**b**) These 90 μm × 90 μm square features can be transferred to the substrate using the same exfoliation process and measured by mapping the Raman G-peak’s center-of-mass.

If features with a precisely defined geometries are desired, a second (optional) patterning of the HOPG block can be performed after the cleaving and before the exfoliation step. In Figure 6a, we show how the HOPG was patterned with 90 μm × 90 μm square features after the cleave, but just before exfoliation. In Figure 6b spatial mapping of the Raman G peak’s center-of-mass after exfoliation confirms the transfer of the 90 μm × 90 μm square feature to a silicon substrate.

## 3. Materials and Methods 

### 3.1. Electrostatic Deposition Setup

**Figure 7:**
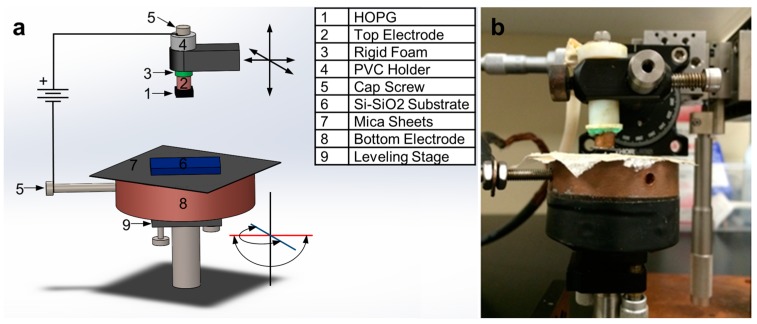
(**a**) Schematic of the electrostatic graphene deposition setup. (**b**) Photograph of the actual experimental setup (outside the glove box).

The electrostatic deposition of graphene starts from a bulk graphitic material. In this case, we use HOPG grade SPI-1 from SPI Supplies^®^ with mosaic angles of 0.4° ± 0.1° and a typical lateral grain size of 3 mm. The substrate used for deposition is a silicon wafer with a 1 µm-thick thermal-oxide layer. In the experimental setup shown in Figure 7, the HOPG block is glued to a top electrode using silver epoxy. The top electrode consists of a 99% pure 1/2″ copper rod cut and polished to 3 cm in length. The top electrode is threaded to accommodate a 1/4″ × 1″ cap screw, which is used to connect the positive terminal of the high-voltage power supply. The top electrode is then placed inside a custom Polyvinyl Chloride PVC holder held by a XYZ micro-positioner stage. The stage can align and bring the HOPG in contact with the deposition substrate at the desired location on the substrate. A 2 mm-thick piece of rigid foam is attached to the bottom of the PVC holder to homogenize the horizontal forces applied on the HOPG block during the contact with the deposition substrate. The bottom electrode consists of a 4 in diameter 99% copper puck of 3 cm in height, also threaded to accommodate a 1/4″ × 1/2″ cap screw to connect the ground terminal of the high-voltage power supply. Three layers of 0.25mm-thick mica sheets are glued to the top of the bottom electrode with AA3106 (Loctite^®^, Dusseldorf, Germany) UV cured PVC bonding adhesive to create an insulating layer between the bottom electrode and the deposition substrate. The bottom electrode is attached to a gimbal levelling stage to ensure that the HOPG and the deposition substrate are parallel to each other so they come into complete contact. A Stanford Research Systems model PS375 +20 kV 10 W power supply is used as the high voltage power supply to connect the top and bottom electrodes with a high voltage cable. The set-up is placed in a Precise Basic Glove Box (Labconco^®^, Kansas City, MO, USA) with a controlled-atmosphere and purged with nitrogen to prevent arcing between the two electrodes.

### 3.2. Pre-Patterning and Cleaving of the HOPG Block

Prior to deposition, the HOPG block is pre-patterned to create an array of square islands using a Samurai UV laser marking system (DPSS Laser Inc, Santa Clara, CA, USA). operating at 355 nm with a spot size of ~25 μm. An etching depth between 0–300 μm can be achieved using this system on HOPG and is precisely controlled via the number of etching laser passes. The etching pattern is drawn using WinLase^®^ (LanMark Controls Inc., Acton, MA, USA) Professional. This process was done originally to limit the size of the deposited graphene; however we will demonstrate it significantly improves the homogeneity of the cleaving process between depositions. Cleaving of the HOPG surface is performed between each deposition using Scotch-tape (MIL-A-AA-113-B, Type 1 Class A, formerly LT-90). On some occasions, the cleaving process completely removes the etched features from the HOPG and leaves a flat surface. 

### 3.3. Deposition and Characterization

Indeed, we find that large-scale deposition of graphene occurs after the complete cleaving of the etched features while using an exfoliation voltage of 3kV with a contact time of 3 s. 

The large-scale depositions of graphene are probed using Raman micro-spectroscopy (Witec, Ulm, Germany) to precisely identify the number of graphene layers within these depositions. A Witec Alpha 300R Raman system is used for the imaging and characterization of the deposited graphene. Raman measurements are performed with a 532 nm excitation laser at a power density of 0.57 mW/μm^2^ using a 10× objective.

Hyperspectral analysis is also performed, both to confirm the Raman results and to analyze the size of the deposition with a larger field of view. For this hyperspectral analysis, a Raman imaging system, RIMA™ NANO (Photon Etc., Montreal, QC, Canada) with an excitation wavelength of 532 nm at a power density of 0.12 mW/μm^2^ is used. A 50× objective is used to show the deposition in its entirety. Further Raman analysis is performed on the HOPG cleaved surface with and without the etched island anchoring process, to emphasize the role of the cleaving method.

**Figure 8:**
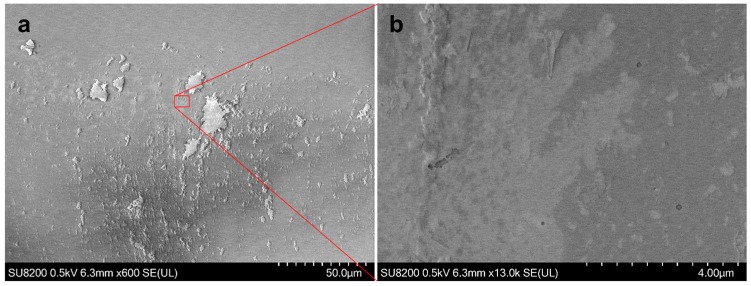
SEM images of the deposited graphene, (**a**) large field of view showing the multi-layered features and the bi-layered “ripped” features. (**b**) Enlarged image showing the ripped bi-layer features as well as the almost transparent monolayer.

SEM micrographs were also taken using a SU 8230 FE-SEM (Hitachi, Rexdale, ON, Canada) with a cold field emission gun to characterize the deposition and confirm the Raman and Hyperspectral layer identification methods. Figure 8a,b shows the deposition in more detail than the optical images previously shown. Similarly to the optical images, the single layered graphene can barely be seen, although it can be distinguished from bi-layered graphene in Figure 3b. It can be seen here that the underlying graphene monolayer is not a single continuous sheet but shows some discontinuity. The same “ripped” features of the bi-layered graphene is even more apparent in these images. Moreover, we can also see some 3 layered graphene features that were not shown on the Raman image in Figure 1c, this is likely due to their small size, the resolution of the Raman image and the 2D peak center method of identification.

## 4. Conclusions

In this report, we demonstrate large-scale graphene deposition, up to 130 μm × 130 μm with 96% coverage (including 92% coverage with single-or double-layer graphene) using electrostatic exfoliation. This is achieved by improving the cleaving process used in preparation for the electrostatic deposition process. Indeed, an array of island features etched into the HOPG substrate seemingly act as anchors to the underlying graphene sheet when scotch tape cleaving is performed, thus significantly improving the homogeneity of the cleaving process as a whole. This critical preparation step is responsible for the superior results reported in this work. We also demonstrate that a second patterning of the HOPG block between the cleave and the exfoliation can be used to achieve features with precisely controlled geometries. We believe this work can have a significant impact as a promising approach to integrating graphene in production of next-generation devices.

## Figures and Tables

**Table 1:** Evolution of FWHM of 2D Raman Peak Lorentzian Fitting from 1 to 5 layered graphene. All FWHM values given in cm^−1^.

Layers/Peaks	2630 (cm^−1^)	2660 (cm^−1^)	2677 (cm^−1^)	2683 (cm^−1^)	2700 (cm^−1^)	2717 (cm^−1^)	2725 (cm^−1^)
Monolayer	-	55	-	-	-	-	-
Bilayer	50	34	30	-	32	-	-
3 layers	26	26	28	28	26	26	-
4 layers	30	25	26	26	25	24	-
5 Layers	30	25	-	26	27	24	26
